# Local Macrophage-Related Immune Response Is Involved in Cochlear Epithelial Damage in Distinct *Gjb2*-Related Hereditary Deafness Models

**DOI:** 10.3389/fcell.2020.597769

**Published:** 2021-01-11

**Authors:** Kai Xu, Sen Chen, Le Xie, Yue Qiu, Xue Bai, Xiao-Zhou Liu, Hui-Min Zhang, Xiao-Hui Wang, Yuan Jin, Yu Sun, Wei-Jia Kong

**Affiliations:** ^1^Department of Otorhinolaryngology, Union Hospital, Tongji Medical College, Huazhong University of Science and Technology, Wuhan, China; ^2^Tongji Medical College, Institute of Otorhinolaryngology, Huazhong University of Science and Technology, Wuhan, China

**Keywords:** immune response, macrophage, *Gjb2*, hereditary deafness, CX3CL1

## Abstract

The macrophage-related immune response is an important component of the cochlear response to different exogenous stresses, including noise, ototoxic antibiotics, toxins, or viral infection. However, the role of the immune response in hereditary deafness caused by genetic mutations is rarely explored. *GJB2*, encoding connexin 26 (Cx26), is the most common deafness gene of hereditary deafness. In this study, two distinct Cx26-null mouse models were established to investigate the types and underlying mechanisms of immune responses. In a systemic Cx26-null model, macrophage recruitment was observed, associated with extensive cell degeneration of the cochlear epithelium. In a targeted-cell Cx26-null model, knockout of Cx26 was restricted to specific supporting cells (SCs), which led to preferential loss of local outer hair cells (OHCs). This local OHC loss can also induce a macrophage-related immune response. Common inflammatory factors, including TNF-α, IL-1β, Icam-1, Mif, Cx3cr1, Tlr4, Ccl2, and Ccr2, did not change significantly, while mRNA of Cx3cl1 was upregulated. Quantitative immunofluorescence showed that the protein expression of CX3CL1 in Deiters cells, a type of SC coupled with OHCs, increased significantly after OHC death. OHC loss caused the secondary death of spiral ganglion neurons (SGNs), while the remaining SGNs expressed high levels of CX3CL1 with infiltrated macrophages. Taken together, our results indicate that CX3CL1 signaling regulates macrophage recruitment and that enhancement of macrophage antigen-presenting function is associated with cell degeneration in Cx26-null mice.

## Introduction

Traditionally, the inner ear has been recognized as an “immune privileged” organ, similar to the eyes or brain. However, there is increasing evidence that immune responses are involved in the inner ear damage process induced by various exogenous stresses, including noise, ototoxic antibiotics, toxins, or viral infection (Fredelius and Rask-Andersen, 1990; Keithley and Harris, 1996; Ladrech et al., 2007; Fujioka et al., 2014; Kaur et al., 2015a). During these cochlear damage processes, macrophages/monocytes are the major immune cells contributing to cochlear immune responses (Hirose et al., 2005, 2014; Sato et al., 2010; Kaur et al., 2015b). The role of macrophages in inner ear damage has not been fully elucidated, and it is generally believed that they are involved in phagocytosis, antigen presentation, or production of immune and inflammatory molecules (Hu et al., 2018). Chemokines and cytokines induced by different stresses are also involved in the regulation of macrophages/monocytes and cochlear pathogenesis (Wood and Zuo, 2017). Moreover, treatments targeting inflammation can effectively alleviate the different types of inner ear damage that have involved an immune response (Canlon et al., 2007; Wakabayashi et al., 2010; Arpornchayanon et al., 2013).

Previous studies on cochlear inflammation mostly focused on acquired deafness caused by exogenous stresses. However, macrophage invasion has also been observed in degenerated stria vascularis in a mouse model of Pendred syndrome, which is a hereditary deafness model caused by mutations in the *SLC26A4* gene (Jabba et al., 2006). This finding indicates that an immune response is associated with hereditary deafness caused by gene mutation, and it raises more questions. Is an immune response involved in other types of hereditary deafness? Will the possible immune response have distinct characteristics due to different deafness genes? Can the inner ear damage of hereditary deafness be alleviated by modulating the immune response? To clarify the above problems, it is necessary to explore the types and characteristics of immune response in more genetic deafness models.

*GJB2* [encoding connexin 26 (Cx26)] is the most common deafness gene, and its mutations are responsible for a quarter of hereditary deafness cases worldwide (Rabionet et al., 2000; Dai et al., 2009). Cx26 assembles with connexin 30 to form gap junctions, which allow small molecules to pass through adjacent supporting cells (SCs) and fibrocytes in the mammalian cochlea (Harris, 2001; Forge et al., 2003; Zhang et al., 2005). In Cx26-null mice, auditory hair cell (HC) and SC deaths with secondary spiral ganglion neuron (SGN) degeneration are the major pathological changes (Sun et al., 2009; Wang et al., 2009; Chen et al., 2014). These pathological phenomena are highly similar to the cochlear damage induced by noise or ototoxic antibiotics, although the underlying mechanism is different (Jiang et al., 2017; Sha and Schacht, 2017). Moreover, HC and SGN deaths induced by noise or ototoxic antibiotics are associated with macrophage recruitment and cochlear inflammation (Kaur et al., 2018; He et al., 2020). Taken together, these observations indicate that macrophage-related inflammation may contribute to cochlear damage in *GJB2*-related deafness.

In this study, two distinct murine models were established to investigate the immune response in a Cx26 knockout. One line was the systemic Cx26-null model, which can induce rapid and significant HC and SC deaths (Chen et al., 2014). The other line was the Cx26-null model of targeted cells, which leads to moderate HC death and delayed SC degeneration. The observation of cochlear macrophages was quantified and compared in these models. Moreover, chemokines and cytokines were analyzed quantitatively. These findings will help us better understand the characteristics and role of the immune response in *GJB2*-related deafness.

## Results

### No Macrophage-Related Immune Response Before Cochlear Cell Death Induced by Knockout of Cochlear Cx26

**FIGURE 1:**
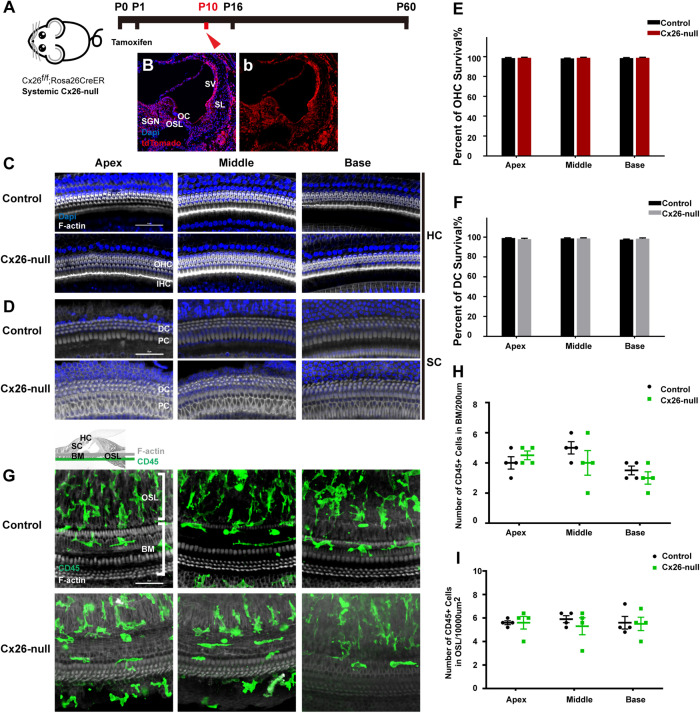
Cell degeneration pattern and distribution of macrophages in the systematic Cx26-null mouse model at P10. **(A)** Cx26^f/f^; Rosa26CreER mice were injected subcutaneously with tamoxifen at P0 and P1 and sacrificed at P10. (**B**,b) tdTomato staining (red) showed that Cre recombinase was activated in cells of the cochlea. **(C,D)** Representative images of HCs and SCs (F-actin, white) in different regions from control and Cx26-null groups. **(E,F)** Quantifications of OHC **(E)** or DC **(F)** survival in different groups. **(G)** Distribution of CD45^+^ cells (green) in the scala tympani side of the BM of different groups. **(H,I)** Comparison of the numbers of CD45^+^ cells in BM **(H)** or OSL **(I)** between control and Cx26-null groups. The scales in **(C,D,G)** represent 50 μm. SGN, spiral ganglion neuron; OC, organ of Corti; OSL, osseous spiral lamina; SV, stria vascularis; SL, spiral ligament; BM, basilar membrane; HC, hair cell; SC, supporting cell.

Cx26^f/f^; Rosa26CreER mice were used to establish a systemic Cx26-null mouse model (Figure 1A). Cochlear Cre activation was visualized by tdTomato expression, and almost all types of cells in the cochlea could be activated (Figures 1B,b). Images of nuclear staining [4′,6-diamidino-2-phenylindole (DAPI), blue] and stereocilia staining (phalloidin, white) were merged to visualize HCs and SCs (Figures 1C,D). At P10, no significant HC or SC loss was observed in the systemic Cx26-null group (Figures 1E,F). CD45 immunostaining (green) was performed to show the distribution pattern of residual macrophages in the scala tympani side of the basilar membrane (BM) at P10 (Figure 1G). The image was captured at the BM level (Figure 1G). Quantification of the results (*n* = 4 mice in each group) showed that there was no significant difference in the number of CD45^+^ cells in all turns of the BM or the osseous spiral lamina (OSL) between the control and systemic Cx26-null groups (Figures 1H,I).

### Recruitment and Activation of Macrophages in Response to Acute Cell Degeneration in Systemic Cx26-Null Mice

**FIGURE 2:**
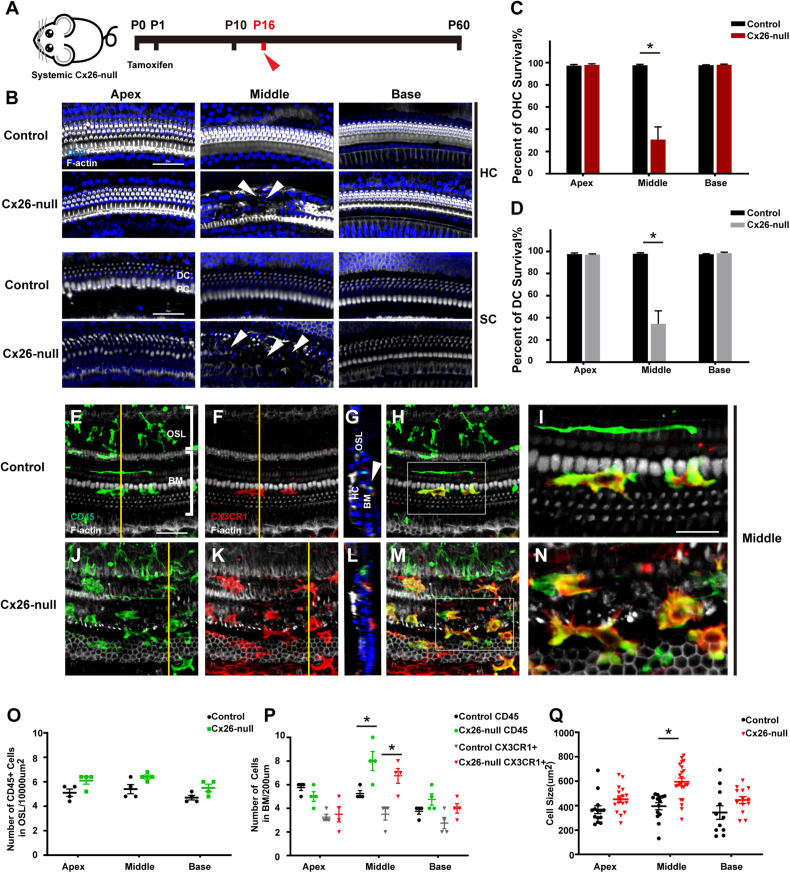
Cell degeneration pattern and distribution of macrophages in the systemic Cx26-null mouse model at P16. **(A)** Systemic Cx26-null mice were sacrificed at P16. **(B)** Representative images of OHCs and DCs (F-actin, white) in different turns from the control and Cx26-null groups. White arrowheads indicate the absence of OHCs and DCs in the middle cochlea of the Cx26-null group. **(C,D)** Quantifications of OHC **(C)** or DC **(D)** survival from different groups. **(E–H)** Double labeling of immune cells with CD45 (green) and CX3CR1 (red) in the middle cochlea of the control group. **(J–M)** Double labeling of immune cells with CD45 (green) and CX3CR1 (red) in the middle cochlea of the Cx26-null group. **(G,L)** Cross-sectional views were generated to show the distribution of macrophages, and yellow lines in **(E,K)** indicate locations of corresponding cross sections. The white boxes in **(H,M)** are magnified in **(I,N)**. **(O)** Comparison of the numbers of CD45^+^ cells in OSL between control and Cx26-null groups. **(P)** Comparison of the numbers of CD45^+^ and CX3CR1^+^ cells on the scala tympani side of the BM between control and Cx26-null groups. **(Q)** Quantifications of macrophage size in different regions from control and Cx26-null groups. The scales in **(B,E)** represent 50 μm. The scale in **(I)** represent 25 μm. *Significant difference from the control group (*p* < 0.05).

Cx26^f/f^; Rosa26CreER mice were used to establish a systemic Cx26-null mouse model (Figure 2A). Consistent with our previous report, significant degeneration of HCs and neighboring SCs was observed in the middle cochlea of the systemic Cx26-null group at P16 (white arrowheads, Figure 2B; Chen et al., 2014). Cell counts (*n* = 4 mice in each group) showed that the proportions of surviving outer hair cells (OHCs) and Deiters cells (DCs, a type of SC coupled with OHCs) were 30.7 ± 11.5% (*p* < 0.0001) and 34.7 ± 11.7% (*p* = 0.0001) in the middle region, while no loss of OHCs or DCs was observed in the apex or base at P16 (Figures 2C,D). Immunostaining of CD45 (green) and CX3CR1 (red) was performed to show the morphology and distribution pattern of macrophages in the scala tympani side of the BM or OSL region (Figures 2E–N). In the area of the BM, cell counts (*n* = 4 mice per group) showed that the number of CD45^+^ cells in the middle region of the systemic Cx26-null group (8.0 ± 0.8) increased significantly compared with that of the control group (5.3 ± 0.3, *p* = 0.0181; Figures 2E–N,P). Cross-sectional views were generated to show the distribution of macrophages (Figures 2G,L, yellow lines in panels E and K indicate locations of corresponding cross sections). Similarly, the number of CX3CR1^+^ cells also increased in this area (3.5 ± 0.5 in the control group and 6.8 ± 0.6 in the Cx26-null group, *p* = 0.0068, Figures 2E–N,P). Moreover, we examined the cell morphology, an essential indicator of macrophage activation. In the systemic Cx26-null group, most of the CD45^+^/CX3CR1^+^ cells near the cochlear injury displayed enlarged cell bodies with dendritic projections, which indicated that they were activated macrophages (Figure 2N). However, some CD45^+^/CX3CR1^+^ cells were round and small, suggesting that they were infiltrated monocytes. Quantification showed that the average size of CD45^+^/CX3CR1^+^ cells in the middle cochlear region of the systemic Cx26-null group (595.2 ± 139.4 μm^2^) was significantly larger than that of the control group (395.5 ± 104.0 μm^2^, *p* < 0.0001, Figure 2Q). No significant difference in the number of CD45^+^ cells in OSL regions was observed between the control and systemic Cx26-null groups (Figure 2O).

### Dynamic Recruitment and Activation of BM Macrophages in Response to Mild OHC Death in Targeted-Cell Cx26-Null Mice

**FIGURE 3:**
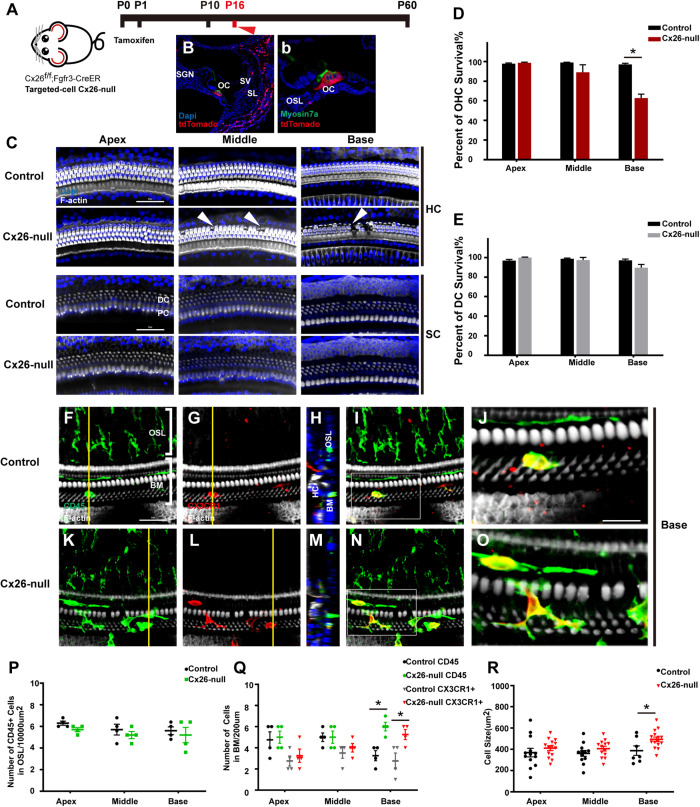
Cell degeneration pattern and distribution of macrophages in the targeted-cell Cx26-null mouse model at P16. **(A)** Cx26^f/f^; Fgfr3iCreERT2 mice were injected subcutaneously with tamoxifen at P0 and P1 and sacrificed at P16. **(B,b)** tdTomato staining (red) showed that Cre recombinase was mainly activated in DCs and PCs. **(C)** Representative images of OHCs and DCs (F-actin, white) in different regions from the control and Cx26-null groups. White arrowheads indicate the absence of OHCs in the middle and basal cochleae of the Cx26-null group. **(D,E)** Quantification of OHC **(D)** or DC **(E)** survival from different groups. **(F–I)** Double labeling of immune cells with CD45 (green) and CX3CR1 (red) in the base of the control group. **(K–N)** Double labeling of immune cells with CD45 (green) and CX3CR1 (red) in the base of the Cx26-null group. **(H,M)** Cross-sectional views were generated to show the distribution of macrophages, and yellow lines in **(G,L)** indicate locations of corresponding cross sections. The white boxes in **(I,N)** are magnified in **(J,O)**. **(P)** Comparison of the numbers of CD45^+^ cells in OSL between control and Cx26-null groups. **(Q)** Comparison of the numbers of CD45^+^ and CX3CR1^+^ cells in the BM between the control and Cx26-null groups. **(R)** Quantifications of macrophage size in different regions from the control and Cx26-null groups. The scales in **(C,F)** represent 50 μm. The scale in **(J)** represent 25 μm. *Significant difference from the control group (*p* < 0.05).

The Cx26^f/f^; Fgfr3iCreERT2 line (Figure 3A) was used to establish a targeted-cell Cx26-null mouse model. The tdTomato signals (red) were mainly observed in DCs and pillar cells (PCs) at P7, which suggests that Cx26 can be knocked out successfully in these cells (Figures 3B,b). In this line at P16, local Cx26 deletion induced OHC loss without corresponding DC death in the base (white arrowheads, Figure 3C). Occasionally, scattered OHC death was observed in the middle cochlear region (white arrowheads, Figure 3C). In targeted-cell Cx26-null mice, quantification (*n* = 4 mice per group) showed that the proportions of surviving OHCs were 98.8 ± 1.2%, 89.2 ± 13.3%, and 62.8 ± 6.9% in the apical, middle, and basal cochlear regions, respectively (Figure 3D). At the same time (P16), no significant DC loss was observed in any turns of this line (Figure 3E). Under normal conditions, macrophages labeled by CD45 and CX3CR1 staining in the base exhibited amoeboid morphology (Figures 3F–J). Cross-sectional views were generated to show the distribution of macrophages (Figures 3H,M). However, macrophages in the cochlear base of the Cx26-null group displayed enlarged cell bodies with multiple protuberances (Figures 3K–O). Quantification (*n* = 4 mice per group) showed that the number of CD45^+^ cells in the basal region of the Cx26-null group increased significantly (6.0 ± 0.7 vs. 3.3 ± 0.8, *p* = 0.0047, Figure 3Q). Similarly, the number of CX3CR1^+^ cells in the base also increased (5.3 ± 0.8 vs. 2.8 ± 1.3, *p* = 0.0307, Figure 3Q). The average size of BM macrophages in the basal region of the Cx26-null group (493.8 ± 80.9 μm^2^) was larger than that of the control group (387.4 ± 108.5 μm^2^, *p* = 0.0239, Figure 3R). No significant difference was observed in the number of CD45^+^ cells in all turns of the OSL region between the control and Cx26-null groups (Figure 3P).

**FIGURE 4:**
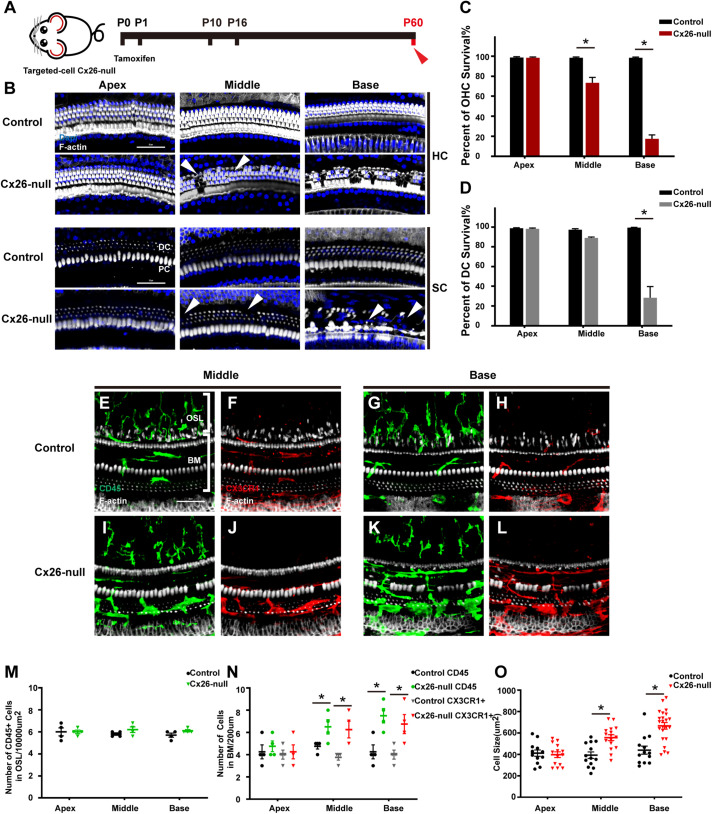
Cell degeneration pattern and distribution of macrophages in the targeted-cell Cx26-null mouse model at P60. **(A)** Targeted-cell Cx26-null mice were sacrificed at P60. **(B)** Representative images of OHCs and DCs (F-actin, white) in different regions from the control and Cx26-null groups. White arrowheads indicate the absence of OHCs or DCs in the middle or basal cochlea of the Cx26-null group. **(C,D)** Quantifications of OHC **(C)** or DC **(D)** survival from different groups. **(E–H)** Double labeling of immune cells with CD45 (green) and CX3CR1 (red) in the middle **(E,F)** and basal **(G,H)** cochleae of the control group. **(I–J)** Double labeling of immune cells with CD45 (green) and CX3CR1 (red) in the middle **(I,J)** and basal **(K,L)** regions of the Cx26-null group. **(M)** Comparison of the numbers of CD45^+^ cells in the OSL between the control and Cx26-null groups. **(N)** Comparison of the numbers of CD45^+^ and CX3CR1^+^ cells in the BM between the control and Cx26-null groups. **(O)** Quantification of macrophage size in different turns from the control and Cx26-null groups. The scales in **(B,E)** represent 50 μm. *Significant difference from the control group (*p* < 0.05).

Mice in the Cx26-null group were sacrificed at P60 (Figure 4A). At P60, OHC loss in the basal turn was further aggravated and extended to the middle cochlea. At the same time, DCs in the base began to disintegrate significantly, and this damage gradually extended to DCs in the middle cochlea (white arrowheads, Figure 4B). Cell counts (*n* = 4 mice per group) showed that the proportions of surviving OHCs were 73.5 ± 9.4% (*p* = 0.0037) or 17.5 ± 6.7% (*p* < 0.0001) in the middle or basal cochlea of the Cx26-null group, respectively (Figure 4C). The proportion of surviving DCs in the base was 28.4 ± 19.6% (*p* = 0.0008, Figure 4D). In the scala tympani side of the BM, most of the macrophages were double positive for CD45 and CX3CR1 (Figures 4E–L). However, the number of macrophages in the OSL area remained unchanged (Figure 4M). The number of CD45^+^ cells in the middle cochlea (6.5 ± 1.1) and the base (7.5 ± 1.1) of the Cx26-null group increased significantly, compared with that of the control group (4.75 ± 0.4 in the middle cochlea, *p* = 0.0447; 4.25 ± 1.1 in the base, *p* = 0.0112, Figure 4N). CX3CR1^+^ cell counts showed similar results (Figure 4N). In the middle cochlea of the Cx26-null group, the mean size of BM macrophages was 556.4 ± 105.6 μm^2^, much larger than that of the control group (372.7 ± 106.0 μm^2^, *p* = 0.0004, Figure 4O). Similarly, macrophages in the base of the Cx26-null group were larger than those in the control group (667.2 ± 144.8 μm^2^ vs. 439.9 ± 129.7 μm^2^, *p* < 0.0001, Figure 4O).

### Increased Number of MHC-2-Positive Cells in the Targeted-Cell Cx26-Null Mice

**FIGURE 5:**
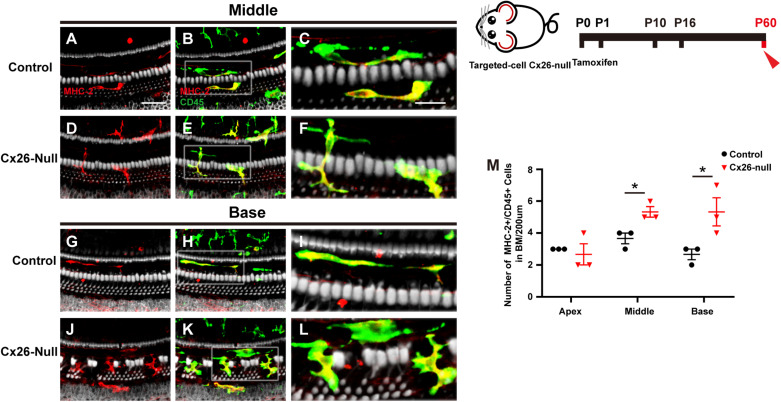
MHC-2 staining in the targeted-cell Cx26-null mouse model at P60. **(A–C)** Double labeling of immune cells with MHC-2 (red, **A**) and CD45 (green, **B**) in the middle region of the control group. **(D–F)** Double labeling of immune cells with MHC-2 (red, **D**) and CD45 (green, **E**) in the middle cochlea of the Cx26-null group. **(G–I)** Double labeling of immune cells with MHC-2 (red, **G**) and CD45 (green, **H**) in the base of the control group. **(J–L)** Double labeling of immune cells with MHC-2 (red, **J**) and CD45 (green, **K**) in the base of the Cx26-null group. The white boxes in **(B,E,H,K)** are magnified in **(C,F,I,L)**, respectively. **(M)** Comparison of the numbers of CD45^+^ and MHC-2^+^ cells in the BM between the control and Cx26-null groups. The scales in **(A,C)** represent 50 and 25 μm, respectively. *Significant difference from the control group (*p* < 0.05).

MHC-2 and CD45 double staining was performed in the targeted-cell Cx26-null mouse model at P60. Most CD45^+^ cells also expressed MHC-2, and a dendritic-to-amoeboid morphology suggested that these CD45^+^/MHC-2^+^ cells were macrophages (Figures 5A–L). The number of CD45^+^/MHC-2^+^ cells in the middle cochlea (5.3 ± 0.3) increased significantly, compared with that of the control group (3.7 ± 0.3, *p* = 0.024, Figure 5M). Similarly, CD45^+^/MHC-2^+^ cells in the base of the Cx26-null group were larger than those in the control group (5.3 ± 0.3 vs. 2.7 ± 0.3, *p* = 0.047, Figure 5M).

### Positive Correlation of the Number of BM Macrophages With the Death Rate of OHCs

**FIGURE 6:**
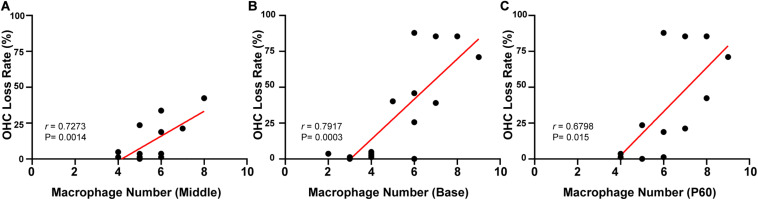
Correlation between the number of BM macrophages and the OHC loss rate in targeted-cell Cx26-null mice. **(A)** Correlation between the number of BM macrophages and OHC loss in the middle cochlea. **(B)** Correlation between the number of BM macrophages and OHC loss in the base. **(C)** Correlation between the number of BM macrophages and OHC loss at P60.

Linear regression was used to analyze the correlation between the number of macrophages and OHC loss. Different turns of the cochlea were analyzed separately. In the targeted-cell Cx26-null line, a significant correlation was found between numbers of BM macrophages and OHC loss in the middle cochlea (*r* = 0.7273, *p* = 0.0014, Figure 6A) and the base (*r* = 0.7917, *p* = 0.0003, Figure 6B). At P60, there were differences in OHC loss in the corresponding turns. Therefore, we conducted a unified analysis of OHC loss in different regions at this time point. Similarly, a positive correlation was found between numbers of BM macrophages and OHC loss at P60 (*r* = 0.6798, *p* = 0.0003, Figure 6C).

### Expression of Cytokines and Chemokines in the Different Cx26-Null Mouse Models

**FIGURE 7:**
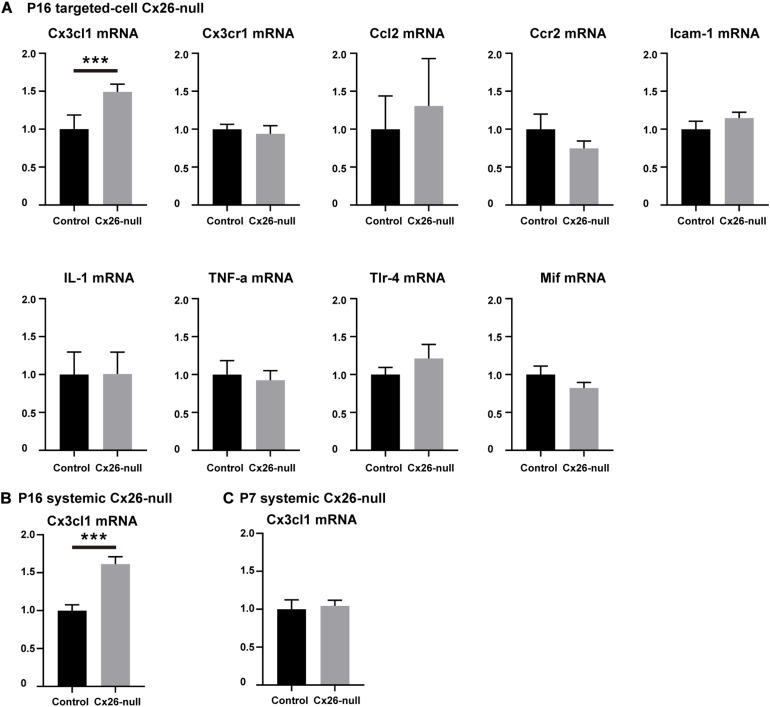
Changes in mRNA expression levels of inflammation-related genes. **(A)** Changes in mRNA expression levels of inflammation-related genes in the cochlea from control and targeted-cell Cx26-null groups at P16 (*n* = 5 in each group). **(B)** Changes in Cx3cl1 mRNA levels in the cochlea from control and systemic Cx26-null groups at P16 (*n* = 6 in each group). **(C)** Changes in Cx3cl1 mRNA levels in the BM from control and systemic Cx26-null groups at P7 (*n* = 6 in each group). ***Significant difference from the control group (*p* < 0.001).

To identify the cytokines and chemokines involved in the macrophage-related immune response of the Cx26-null mouse, a group of mRNAs of inflammation-related genes (Ccl2, TNF-α, IL-1β, Tlr4, Cx3cl1, Mif, Cx3cr1, Ccr2, and Icam-1) was quantitatively analyzed. At P16, a small amount of OHC loss can cause macrophage recruitment in the targeted-cell Cx26-null line. Therefore, we prioritized detection of the cochlear mRNA of this model at this time point. Compared with those in the control group, the mRNA levels of Ccl2, Ccr2, TNF-α, IL-1β, Tlr4, Cx3cr1, Mif, and Icam-1 in the Cx26-null group were not significantly different. However, the mRNA of Cx3cl1 was significantly increased (*n* = 5 per group, *p* = 0.0009, Figure 7A). To further verify our results, Cx3cl1 mRNA from the cochleae of the systemic Cx26-null mice was also quantified. At P16, the Cx3cl1 mRNA level of the systemic Cx26-null group increased by 61.4 ± 9.8% compared with the control group (*n* = 6 in each group, *p* = 0.0006, Figure 7B). P7 is the last time point at which the entire BM can be completely dissected. Considering the recruitment of macrophages mainly occurs at the scala tympani side of the BM, we dissected the BM tissue of this line and extracted mRNA at P7. However, the mRNA level of Cx3cl1 showed no significant difference between the experimental and control groups at P7 (*n* = 6 in each group, *p* = 0.76, Figure 7C).

### Dynamic Changes in the Expression Level of CX3CL1 in the Cochlea and the Number of Macrophages in the Rosenthal Canal of Cx26-Null Mice

**FIGURE 8:**
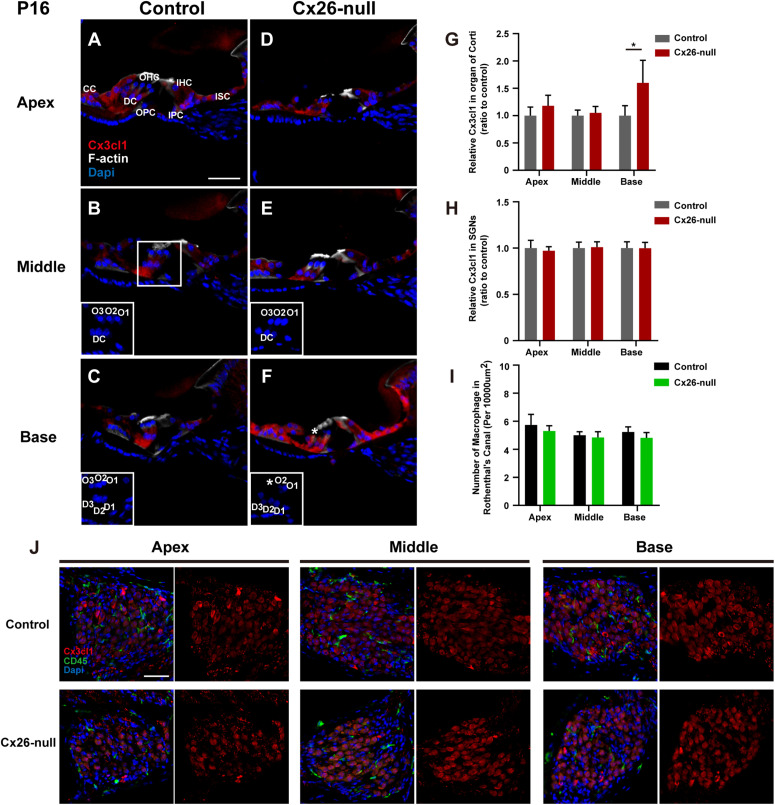
Quantitative expression level of CX3CL1 in the OC and distribution of macrophages in the RC region from control and targeted-cell Cx26-null groups at P16. **(A–C)** Modiolar sections showed immunolabeling of CX3CL1 (red) of OCs in different regions from the control group. **(D–F)** Parallel images of CX3CL1 immunostaining in different regions from the targeted-cell Cx26-null group. The white boxes at the bottom right of the **(C–F)** show the DAPI staining in OCs, and * indicates missing hair cell. **(G,H)** Quantification of CX3CL1 immunostaining in OCs (G) or SGNs **(H)** in the different groups at P16 (*n* = 4 mice in each group). **(I)** Quantification of macrophage numbers per 10,000 μm^2^ of the RCs in different groups at P16 (*n* = 4 mice in each group). **(J)** Modiolar sections showed macrophages (CD45, green) and CX3CL1 (red) immunolabeling in RC regions from different groups. *Significant difference from the control group (*p* < 0.05). O1, OHC1; D1, DC1; IPC, inner pillar cell; OPC, outer pillar cell; ISC, inner sulcus cell; CC, Claudius cell.

To provide further evidence of CX3CL1 expression, CX3CL1 immunostaining (red) was performed to quantify the expression of CX3CL1 in the organ of Corti (OC) and the Rosenthal canal (RC). Consistent with a previous report, CX3CL1 was expressed in cells of the OC, including DCs, PCs, inner hair cells (IHCs), and other SCs (Figure 8A). However, there was almost no CX3CL1 expression in OHCs (Kaur et al., 2015b). Compared with the control group, there was no significant difference in the expression of CX3CL1 in apical (Figures 8A,D) and middle (Figures 8B,E) cochleae of the OCs in the Cx26-null group at P16. However, CX3CL1 expression in the basal turn of the OC increased significantly in Cx26-null mice (Figures 8C,F, ^∗^ indicates the missing OHC). In the OC with OHC loss, the expression of CX3CL1 in DCs increased significantly (Figure 8F). To quantitatively analyze CX3CL1 expression in the OC, an area of the same size including DCs, OHCs, PCs, and IHCs was selected and analyzed (three to four sections per mouse, *n* = 4 mice in each group). This showed that the relative fluorescence density in the basal OC from the Cx26-null group increased by 60.1 ± 40.1% (*p* = 0.032, Figure 8G). In the RC, CX3CL1 was mainly expressed in the bodies of SGNs. However, the relative fluorescence density of CX3CL1 staining in the SGNs of the Cx26-null group did not change significantly at P16 (Figure 8H). Additionally, there was no significant difference in the number of macrophages (green) in any turns of the RC between the control and Cx26-null groups at P16 (Figures 8J,I).

**FIGURE 9:**
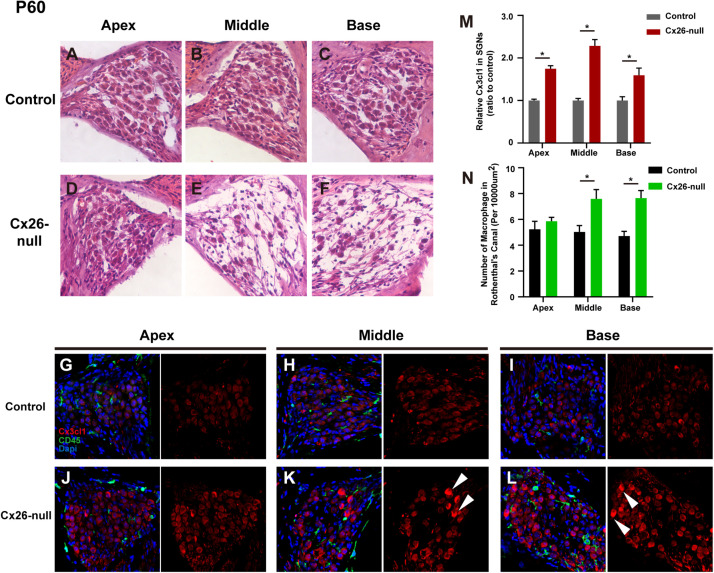
SGN loss pattern and distribution of macrophages in the RC region from control and targeted-cell Cx26-null groups at P60. **(A–C)** Representative images of SGN in the apex **(A)**, middle region **(B)**, and base **(C)** from the control group at P60. **(D–F)** Parallel images in different regions from the targeted-cell Cx26-null group at P60. **(G–I)** Macrophages (CD45, green) and CX3CL1 (red) immunolabeling in the apex, middle region, and base of the control group, respectively. **(J–L)** Macrophages (CD45, green) and CX3CL1 (red) immunolabeling in corresponding regions of the targeted-cell Cx26-null group. White arrowheads indicate the SGN with high expression of CX3CL1. **(M)** Quantification of CX3CL1 fluorescence in SGNs in the different groups at P60 (*n* = 4 mice in each group). **(N)** Quantification of macrophage numbers per 10,000 μm^2^ of RCs in the different groups at P60 (*n* = 4 mice in each group). *Significant difference from the control group (*p* < 0.05).

At P60, significant cellular degeneration of the SGN was observed in the middle and basal cochleae of the Cx26-null group (Figures 9A–F). Accordingly, an increase in the number of macrophages was observed in the corresponding area (Figures 9G–L). Quantification of the results (*n* = 4 mice in each group) showed that the number of CD45^+^ cells per 10,000 μm^2^ of RCs in the middle (7.6 ± 0.2) and basal cochleae (7.7 ± 0.6) from the Cx26-null group increased significantly compared with that of the control group (5.0 ± 0.5 in the middle turn, *p* = 0.0068; 4.7 ± 0.36 in the basal turn, *p* = 0.0003, Figure 9N). At the same time, quantification (two to three sections per mouse, *n* = 4 mice in each group) showed that the relative fluorescence of CX3CL1 in the apical, middle, or basal cochlea of SGNs from the Cx26-null group increased by 74.9 ± 6.9% (*p* < 0.0001), 128.6 ± 14.8% (*p* < 0.0001) or 59.3 ± 16.9% (*p* = 0.0059), respectively (Figure 9M). In particular, some SGNs of the middle region and base from the experimental group displayed strong expression of CX3CL1 at P60 (arrowheads, Figures 9K,L).

## Discussion

### The Macrophage-Related Immune Response Is Involved in the Degeneration Process of Cochlear Sensory Epithelial Cells Induced by Cx26 Deletion

In a previous study, CD45 labeling and morphological observation were used to identify macrophages/monocytes in a mouse model of noise-induced deafness. The majority of CD45^+^ cells expressed CX3CR1, while approximately 90% of CX3CR1 cells were CD68- and Iba-1 positive. This discovery allowed the authors to determine that these quad-marker-positive cells were cochlear macrophages (Hirose et al., 2005). Therefore, CD45 and CX3CR1 double labeling combined with morphological observation was used to identify macrophages in our study. Our and other studies showed that systemic knockout of cochlear Cx26 can induce developmental disorders of SCs at P9 (Wang et al., 2009; Chen et al., 2018a). There were no significant changes in the number and morphology of macrophages in the systemic Cx26-null line at P10. Since there was no significant cell death at this time point, we speculated that the recruitment of macrophages has nothing to do with disorders of SC development. In the control group of this line at P16, CD45^+^/CX3CR1^+^ cells were irregular in shape with dendritic projections, suggesting that they were residual macrophages. In the middle cochlea of systemic Cx26-null mice at P16, CD45^+^/CX3CR1^+^ cell numbers were increased in the scala tympani side of the BM where HC and SC deaths occurred. Some of the CD45^+^/CX3CR1^+^ cells were round and small without dendritic projections, suggesting that they were infiltrated monocytes. Some other CD45^+^/CX3CR1^+^ cells showed enlarged bodies with short dendritic projections, which is a morphology typical of macrophages. Obviously, this phenomenon indicated a transforming continuum between infiltrated monocytes and macrophages. Similar changes were observed in the targeted-cell Cx26-null line at P16. Taken together, the pattern of macrophage-related immune responses is consistent with that of cochlear cell degeneration induced by Cx26 knockout.

### OHC Death Directly Activates the Recruitment of Macrophages

In the systemic Cx26-null mice, rapid HC and SC degeneration can trigger the recruitment of macrophages. In the targeted-cell Cx26-null mice, knockout of cochlear Cx26 is limited to PCs and DCs. DCs, a type of SCs coupled with OHCs, are believed to supply nutrients and generate a unique electrochemical environment for OHCs. Once Cx26 expression of a row of DCs is knocked out, the corresponding OHCs will die first in the base (Chen et al., 2018b). In the targeted-cell Cx26-null mice, the number of macrophages increased in the scala tympani side of the BM, where OHCs began to degenerate without SC loss at P16. This indicated that local OHC death is enough to induce recruitment and activation of macrophages. In a previous study, cochlear HCs (including IHCs and OHCs) were specifically killed by diphtheria toxin with no evident pathology among SCs, which also led to an increase in the number of macrophages (Kaur et al., 2015b). Moreover, aminoglycoside-induced OHC death was sufficient to recruit macrophages into the cochlear epithelium (Kaur et al., 2018). It should be noted that OHC degeneration of the targeted-cell Cx26-null line may have been caused by DC dysfunction, because Cx26 was not expressed in OHCs. This is completely different from the direct killing of HCs by toxins or aminoglycoside. Although the mechanism of HC death is completely different between our and their studies, a macrophage-related immune response occurred in these models. This suggested that macrophages/monocytes are highly sensitive to HC damage, especially OHCs.

### In the *Gjb2*-Related Deafness Model, CX3CL1 (Fractalkine) Expressed by the SCs or SGNs Is Involved in Macrophage-Related Inflammation

In previous studies, immune-related gene expression was investigated in cochleae subjected to different types of damage. In noise-induced deafness, proinflammatory cytokines or chemokines, including TNF-α, IL-1β, IL-6, Icam-1, and Ccl2, were increased in cells of the stria vascularis or lateral wall (Fujioka et al., 2006; Tornabene et al., 2006; Tan et al., 2016). In an acute cochlear dysfunction model induced by mitochondrial toxin, high expression of the chemokine genes Ccl1-5, Ccr2, Ccr5, and Cx3cr1 was detected in the lateral wall (Fujioka et al., 2014). However, in the targeted-cell Cx26-null mouse model, only the mRNA level of Cx3cl1 was upregulated. Although macrophage-related inflammation occurred in the above models, the cytokines or chemokines involved are quite different. In noise- and mitochondrial toxin-induced models of cochlear damage, damage to the inner ear is more widespread, including the stria vascularis, lateral wall, and cochlear sensory epithelium. The damage to fibrocytes of the lateral wall is more likely to lead to changes in cytokines or chemokines such as TNF-α, IL-1β, IL-6, and Icam-1 (Fujioka et al., 2006; Tornabene et al., 2006; Fujioka et al., 2014). Extensive knockout of cochlear Cx26 does not cause acute and obvious cell damage to the lateral wall or stria vascularis (Zhou et al., 2016). In contrast, OHC loss is the earliest cell damage in targeted-cell Cx26-null mice at P16, and secondary SGN death occurs at P60. Immunostaining results suggested that CX3CL1 is significantly increased in DCs of damaged OCs at P16, while upregulation of CX3CL1 expression was seen in SGNs at P60. This indicated that the increased expression of CX3CL1 in DCs or SGNs may be directly induced by death of OHCs or SGNs, respectively. However, we do not yet know which signal causes the upregulation of CX3CL1 in SCs. The CX3CL1–CX3CR1 axis has a strong effect on macrophage/monocyte recruitment, and this effect has been observed in fat tissues, the spine, and bones (Han et al., 2014; Polyak et al., 2016; Shen et al., 2018). In injured cochleae, disruption of CX3CL1–CX3CR1 signaling modulates the number of macrophages (Sato et al., 2010; Kaur et al., 2015b). Treatment with anti-CX3CL1 mAb (a neutralizing antibody) can prevent macrophage migration into the synovium of rheumatoid arthritis (RA) in a murine model, and humanized anti-CX3CL1 mAb has been used to treat patients with RA (Nanki et al., 2004; Tanaka et al., 2018). Taken together, these data suggest that the recruitment and activation of macrophages may be caused by high expression of CX3CL1 in damaged cochlear regions of Cx26-null mice. Moreover, lack of CX3CR1 in macrophages enhances synaptic degeneration or SGN loss following ototoxic or acoustic injury (Kaur et al., 2018, 2019). Depletion of macrophages attenuates HC loss and hearing impairment (Mizushima et al., 2017). In the central nervous system, CX3CL1 is released from neurons and interacts with CX3CR1^+^ microglia. A previous study showed that CX3CL1 signaling induces a neuroprotective state in neurons (Noda et al., 2011; Mattison et al., 2013). However, genetic deficiency of CX3CR1 is associated with potentially detrimental secretion of proinflammatory cytokines causing neurotoxicity (Cardona et al., 2006). These data suggest that regulating macrophage function through CX3CL1–CX3CR1 signaling is an effective means to reduce damage to the inner ear.

### The CX3CL1–CX3CR1 Axis Participates in Clearing the Debris of Dead OHCs by Regulating DCs and Macrophages

Macrophage recruitment in the Cx26-null mouse model is a process of sterile inflammation. Without a pathogen, macrophages are recruited to clear debris or facilitate wound healing. In the targeted-cell Cx26-null model, the injured SGNs caused by OC damage directly express CX3CL1 to recruit macrophages. These infiltrated CX3CR1^+^ macrophages promote the survival of SGNs (Kaur et al., 2015b). In the injured utricle, macrophages appear to be actively engulfing HC debris, suggesting that macrophages participate in the process of “corpse removal” in the mammalian vestibular organs and may have a role in promoting tissue repair (Kaur et al., 2015a). However, macrophages are blocked by the BM and cannot directly contact the dead OHCs in mature cochleae (Dong et al., 2018). In ototoxic drug models, SCs can completely engulf the dead bodies of HCs, a behavior highly similar to the phagocytosis of macrophages (Monzack et al., 2015). The signals of dead OHCs can induce high expression of CX3CL1 in DCs of the targeted-cell Cx26-null model. Our investigation indicated that CX3CR1^+^ macrophages may be regulated by DCs through the CX3CL1–CX3CR1 axis, and increased CX3CR1^+^ macrophages and DCs together participate in OHC debris clearance. This corpse removal may promote cochlear repair. However, the specific mechanism and the role of macrophages in the phagocytosis of DCs are still unclear.

In summary, macrophage-related inflammation is involved in the process of cell damage caused by Cx26 knockout. OHC loss caused by Cx26 knockout can recruit and activate macrophages at the scala tympani side of the BM. The death of OHCs can induce DCs to highly express the chemokine CX3CL1, which then participates in the regulation of macrophages through the CX3CL1–CX3CR1 axis.

## Materials and Methods

### Mouse Models

Cx26^loxP/loxP^ (Cx26^f/f^) mice and Rosa26CreER mice were provided by Prof. Xi Lin at Emory University. ROSA26CAG-loxP-stop-loxP-tdTomato (tdTomato) mice were provided by Prof. Ren-Jie Chai at Southeast University. Fgfr3iCreERT2 mice were provided by Prof. Zhi-Yong Liu at the Chinese Academy of Sciences. As reported previously, tamoxifen-inducible Cx26-null mice were generated by crossbreeding of the Cx26^f/f^ mice with Rosa26CreER or Fgfr3iCreERT2mice. Cx26^f/f^; Rosa26CreER and Cx26^f/f^; Fgfr3iCreERT2 mice were maintained on a CBA background. We transferred these mice onto a CBA genetic background by backcrossing for more than four generations. Mouse genotyping was performed by PCR amplification of tail genomic DNA, and the genotyping primers were as follows:

Cx26 (F): 5′-ACAGAAATGTGTTGGTGATGG-3′,
Cx26 (R): 5′-CTTTCCAATGCTGGTGGAGTG-3′,
Rosa26CreER (F): 5′-AGCTAAACATGCTTCATCGTCG GTC-3′,
Rosa26CreER (R): 5′-TATCCAGGTTACGGATATAGTTC ATG-3′,
Fgfr3iCreER (F): 5′-GAGGGACTACCTCCTGTACC-3′,
Fgfr3iCreER (R): 5′-TGCCCAGAGTCATCCTTGGC-3′,
tdTomato-wild-type (F): 5′-AAGGGAGCTGCAGTGG AGT-3′,
tdTomato-wild-type (R): 5′-CCGAAAATCTGTGGGAA GTC-3′,
tdTomato-mutant (F): 5′-GGCATTAAAGCAGCGTATC-3′,
tdTomato-mutant (R): 5′-CTGTTCCTGTACGGCATGG-3′.

In this study, all mice were injected with tamoxifen (T5648-1G, Sigma-Aldrich, St. Louis, MO, United States) subcutaneously at P0 and P1 (0.75 mg/10 g body weight, once a day for 2 consecutive days). The operation procedure for the use of tamoxifen followed the chemical use guidelines of Huazhong University of Science and Technology. The preparation of tamoxifen was carried out in a fume hood, with the operator wearing masks and gloves for self-protection, and waste disposal was regulated. Consistent with a previous study, cochlear Cx26 was extensively knocked out in different types of cells in Cx26^f/f^; Rosa26CreER mice (Chen et al., 2018a). However, Cx26^f/f^; Fgfr3iCreERT2 mice exhibited Cx26 knockout in specific cells of the inner ear. Cx26^f/f^; Rosa26CreER and Cx26^f/f^; Fgfr3iCreERT2 mice were used in the experimental groups, while their littermates without Cre were used as controls.

All mice were raised in the specific-pathogen-free Experimental Animal Center of Huazhong University of Science and Technology. All experimental procedures were conducted in accordance with the policies of the Committee on Animal Research of Tongji Medical College, Huazhong University of Science and Technology.

### Cochlear Tissue Preparation and Immunofluorescent Labeling

Mice (*n* = 4 in each group) were deeply anesthetized and sacrificed at P10, P16, and P60. The cochleae were carefully dissected from the temporal bones and fixed in 4% paraformaldehyde in 0.01 M phosphate-buffered saline (PBS) at room temperature for 1 h. For frozen sections, after decalcification with disodium EDTA for 48 h, the cochleae were dehydrated with 20 and 30% sucrose for 1.5 h each and embedded in optimal cutting temperature compound (OCT) overnight at 4°C. Modiolar sections with a thickness of 10 μm were cut for subsequent procedures. For flattened cochlear preparations, each stretched cochlear preparation was carefully dissected in ice-cold 0.01 M PBS. The sections or flattened cochlear preparations were incubated in a blocking solution (10% donkey serum with 0.1% Triton X-100) for 1 h at room temperature. Samples were then incubated overnight at 4°C with a goat anti-CD45 polyclonal antibody (1:100 dilution, AF114, R&D Systems, Minneapolis, MN, United States), a rabbit polyclonal anti-CX3CR1 antibody (1:200 dilution, NBP1-76949, Novus Biologicals, Littleton, CO, United States), a rabbit polyclonal anti-CX3CL1/fractalkine antibody (1:200 dilution, NBP1-49539, Novus Biologicals), or a rat anti-MHC Class II monoclonal antibody (1:100 dilution, ab25333, Abcam, Cambridge, MA, United States), each diluted in 0.01 M PBS with 0.3% Triton X-100. Samples were washed three times in 0.01 M PBS with 0.1% Tween-20 and then stained with Alexa Fluor 647 donkey anti-goat IgG or Alexa Fluor 488 donkey anti-rabbit IgG (1:200 dilution, ANT032 and ANT031, Antgene, China) for 2 h. DAPI (C1005, Beyotime Biotechnology) and phalloidin (0.05 mg/ml, P5282, Sigma-Aldrich) were used for nuclear and F-actin staining. Images were obtained with a laser scanning confocal microscope (Nikon, Tokyo, Japan). Visualization of macrophages was achieved using CD45/CX3CR1 immunostaining. A total number of approximately 15 macrophages from one mouse were used for quantitative analysis (*n* = 4 mice in each group). The size of BM macrophages was measured using ImageJ software. The immunolabeling of CX3CL1 was quantified from original images, each taken at ×60 magnification under identical conditions. For CX3CL1 expression in the OC, the same size analysis range was used in each turn of the cochleae, and this range mainly included OHCs and DCs (three to four sections per mouse, four mice per group). To quantify the CX3CL1 expression of SGNs, 10 SGNs in an RC region were randomly selected (two to three sections per mouse, four mice per group). Relative fluorescence was quantified by normalizing the ratio of average fluorescence of target cells in the Cx26-null group to that in the control group.

### Macrophage Counts

Visualization of macrophages was achieved using CD45 immunostaining. To assess macrophages per 200 μm of sensory epithelium, CD45-labeled (or CX3CR1-labeled) macrophages were counted from 60 × images taken from the apex, middle, and base of the BM (*n* = 4 mice in each group). To assess the macrophages in the spiral ganglia, CD45-labeled macrophages were counted in 60 × images taken from the apical, middle, and basal portions of RC of sectioned specimens. Macrophages in the spiral ganglia were counted in at least three to four sections per cochlea and reported as number per 10,000 μm^2^ (*n* = 4 mice in each group).

### Quantification of Cochlear HCs and DCs

To assess the patterns of damage to sensory cells, we quantified the number of surviving OHCs and DCs in the apical, middle, and basal turns of stretched cochlear preparations. After permeabilization with 0.3% Triton X-100 in 0.01 M PBS for 15 min, the samples were incubated with phalloidin (0.05 mg/ml, P5282, Sigma-Aldrich) for 30 min at room temperature. Tissues were washed three times in 0.01 M PBS with 0.1% Tween-20, and then nuclei were counterstained with DAPI (C1005, Beyotime Biotechnology). Images at 60 × magnification were captured with a laser scanning confocal microscope in different regions of the flattened preparations (Nikon). A total number of approximately 80 OHCs and DCs from each turn were taken for counting (*n* = 4 mice in each group).

### RNA Preparation and Real-Time Quantitative Polymerase Chain Reaction

RT-qPCR was performed to determine the transcriptional expression level of the following genes: TNF-α, IL-1β, Cx3cl1, Cx3cr1, Ccl2, Ccr2, Icam-1, Tlr4, and Mif. After the animals were euthanized and killed, the membranous labyrinths of the cochleae were dissected carefully on ice. The membranous labyrinth tissue from one cochlea was used to generate one sample. There were six biological replicates for each experimental condition. Total RNA was extracted from the collected tissues using an RNAprep Pure Tissue Kit (Tiangen Biotech Co. Ltd, Beijing, China) and was reverse transcribed using a PrimeScript RT Reagent Kit with gDNA eraser (Takara Bio Inc., Shiga, Japan). Real-time PCR was performed using SYBR Green PCR Technology in a Roche LightCycler 480 instrument. Analysis of relative gene expression data between sample groups was performed according to the standard 2^–ΔΔCP^ method. The following primers were used for RT-qPCR:

TNF-α (F): 5′-CAGGCGGTGCCTATGTCTC-3′
TNF-α (R): 5′-CGATCACCCCGAAGTTCAGTAG-3′
IL-1β (F): 5′-GAAATGCCACCTTTTGACAGTG-3′
IL-1β (R): 5′-TGGATGCTCTCATCAGGACAG-3′
Cx3cl1 (F): 5′-CTGGCCGCGTTCTTCCATT-3′
Cx3cl1 (R): 5′-GCACATGATTTCGCATTTCGT-3′
Cx3cr1 (F): 5′-GAGTATGACGATTCTGCTGAGG-3′
Cx3cr1 (R): 5′-CAGACCGAACGTGAAGACGAG-3′
Ccl2 (F): 5′-TAAAAACCTGGATCGGAACCAAA-3′
Ccl2 (R): 5′-GCATTAGCTTCAGATTTACGGGT-3′
Ccr2 (F): 5′-ATCCACGGCATACTATCAACATC-3′
Ccr2 (R): 5′-TCGTAGTCATACGGTGTGGTG-3′
Icam-1 (F): 5′-GTGATGCTCAGGTATCCATCCA-3′
Icam-1 (R): 5′-CACAGTTCTCAAAGCACAGCG-3′
Tlr4 (F): 5′-ATGGCATGGCTTACACCACC-3′
Tlr4 (R): 5′-GAGGCCAATTTTGTCTCCACA-3′
Mif (F): 5′-GAGGGGTTTCTGTCGGAGC-3′
Mif (R): 5′-GTTCGTGCCGCTAAAAGTCA-3′

### Data Analysis

All data are presented as means ± SE and plotted using GraphPad Prism (Version 8.2.1, GraphPad Software Inc., La Jolla, CA, United States). The *t*-tests or correlation analyses were performed in SPSS software (Version 19, IBM SPSS Statistics, IBM Corp., Armonk, NY, United States), and *p* < 0.05 was considered to be statistically significant.

## Data Availability Statement

The raw data supporting the conclusions of this article will be made available by the authors, without undue reservation, to any qualified researcher.

## Ethics Statement

The animal study was reviewed and approved by the Union Hospital of Tongji Medical College, Huazhong University of Science and Technology. Written informed consent was obtained from the owners for the participation of their animals in this study.

## Author Contributions

YS and W-JK conceived and designed the study and reviewed and edited the manuscript. KX, SC, LX, YQ, XB, X-ZL, H-MZ, X-HW, and YJ performed the experiments. SC and KX wrote the manuscript. All authors read and approved the manuscript.

## Conflict of Interest

The authors declare that the research was conducted in the absence of any commercial or financial relationships that could be construed as a potential conflict of interest.

## Supplementary Material

Supplementary Figure 1 Cell degeneration pattern and distribution of macrophages in the target-cell Cx26-null mouse model at P16 or P60 (cochlear stretched preparation). **(A–D)** Representative images of macrophages (CD45 labeling, green) in cochlear stretched preparations from the control **(A,B)** and the targeted cell Cx26-null group **(C,D)** at P16. (a–d) Representative images of HCs in cochlear stretched preparations from the control (a,b) and the targeted cell Cx26-null group (c,d) at P16. Layers of scanned images are illustrated in the upper left corner of **(A)** and (a). **(E–H)** Representative images of macrophages (CD45 labeling, green) in cochlear stretched preparations from the control **(E,F)** and targeted cell Cx26-null group **(G,H)** at P60. (e–h) Representative images of HCs in cochlear stretched preparations from the control (e,f) and the targeted cell Cx26-null group (g,h) at P60.

## Abbreviations

Cx26: connexin 26
SC: supporting cell
DC: Deiters cell
IPC: inner pillar cell
OPC: outer pillar cell
HC: hair cell
OHC: outer hair cell
IHC: inner hair cell
OC: organ of Corti
SGN: spiral ganglion neuron
OSL: osseous spiral lamina
RC: Rosenthal canal
ISC: inner sulcus cell
CC: Claudius cell.
