# Supplementary figures and images for: Local Macrophage-Related Immune Response Is Involved in Cochlear Epithelial Damage in Distinct Gjb2-Related Hereditary Deafness Models
Source: Front Cell Dev Biol. 2021 Jan 11;8:597769. doi: 10.3389/fcell.2020.597769 (PMC7829512; doi:10.3389/fcell.2020.597769)

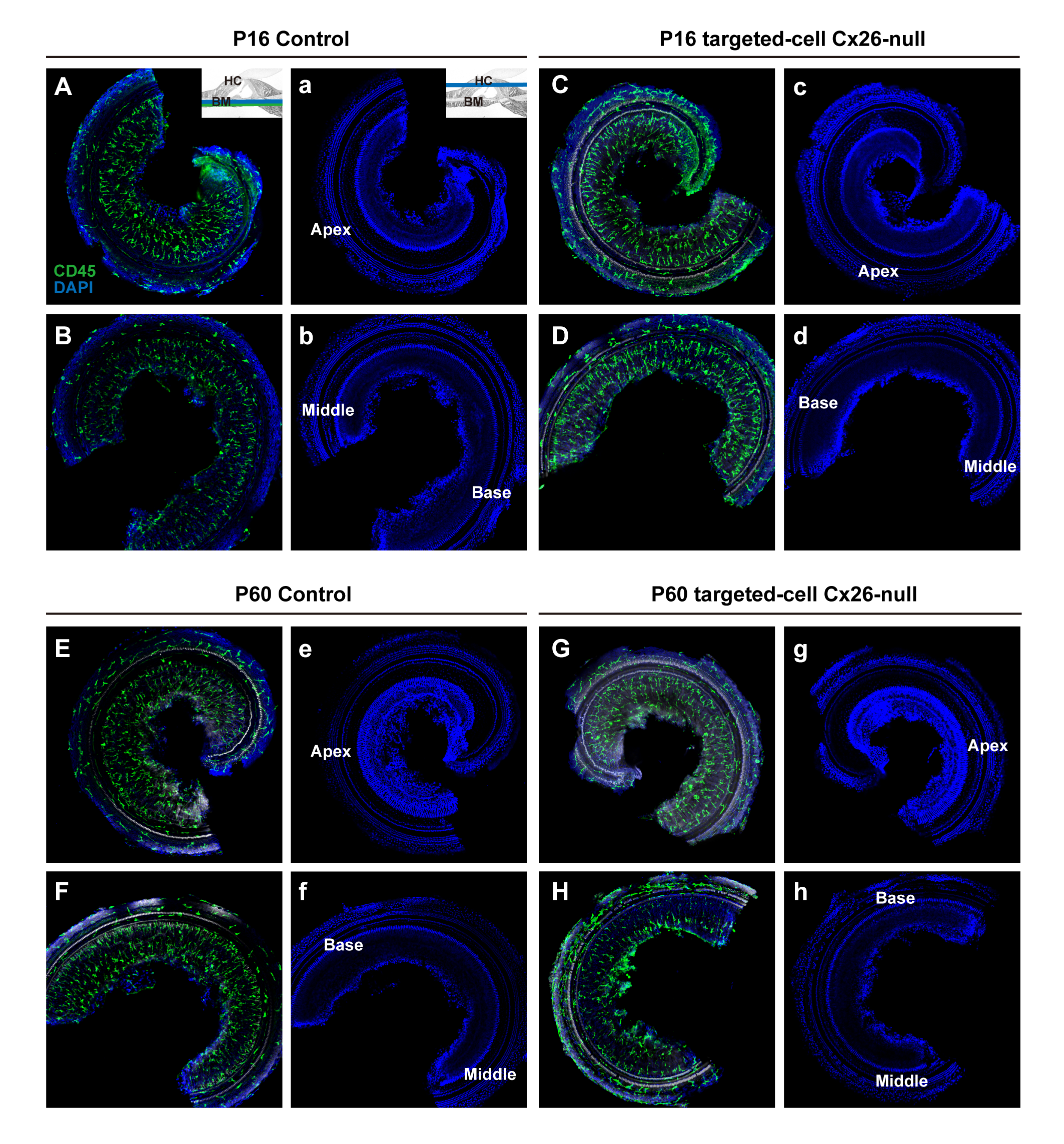

Supplement: Supplementary Figure 1 — Cell degeneration pattern and distribution of macrophages in the target-cell Cx26-null mouse model at P16 or P60 (cochlear stretched preparation). (A–D) Representative images of macrophages (CD45 labeling, green) in cochlear stretched preparations from the control (A,B) and the targeted cell Cx26-null group (C,D) at P16. (a–d) Representative images of HCs in cochlear stretched preparations from the control (a,b) and the targeted cell Cx26-null group (c,d) at P16. Layers of scanned images are illustrated in the upper left corner of (A) and (a). (E–H) Representative images of macrophages (CD45 labeling, green) in cochlear stretched preparations from the control (E,F) and targeted cell Cx26-null group (G,H) at P60. (e–h) Representative images of HCs in cochlear stretched preparations from the control (e,f) and the targeted cell Cx26-null group (g,h) at P60. [file Image_1.TIF]
